# Impact of Apolipoprotein E4 on blood-brain barrier integrity in target replacement murine models: a systematic review and meta-analysis

**DOI:** 10.1186/s13195-026-02018-3

**Published:** 2026-05-07

**Authors:** Krystal K. Laing, Nela Fialova, Joanna Wardlaw, Axel Montagne

**Affiliations:** 1 UK Dementia Research Institute at the University of Edinburgh, 49 Little France Crescent, Edinburgh, EH16 4SB UK; 2 UK Dementia Research Institute Centre for Vascular Dementia Research, British Heart Foundation, University of Edinburgh, Edinburgh, UK; 3 Institute for Neuroscience and Cardiovascular Research (INCR), University of Edinburgh, Chancellor’s Building, Edinburgh, UK

**Keywords:** Apolipoprotein E4, Blood–brain barrier, Cerebral blood flow, Vascular dysfunction, Alzheimer’s disease, Systematic review, Meta-analysis

## Abstract

**Background:**

The E4 variant of Apolipoprotein E (APOE) is a primary genetic susceptibility risk factor for late-onset Alzheimer’s disease and has been implicated in cerebrovascular dysfunction. Preclinical mouse models are widely used to study APOE4, but cohesive understanding of APOE’s role is still inconsistent and lacking. The aim of this study was to systematically review and synthesise evidence from preclinical mouse studies assessing APOE4 related effects on blood-brain barrier (BBB) integrity, vascular morphology and cerebral blood flow (CBF).

**Main:**

A systematic search of MEDLINE, Embase, Scopus, and Web of Science was conducted (March-April 2025). Eligible studies included transgenic APOE-targeted replacement or knock-in mice reporting vascular outcomes (cerebral blood flow, blood brain barrier permeability, vascular measures). Risk of bias was assessed using SYRCLE and reporting quality with CAMARADES. Random-effects meta-analyses were conducted (where sufficient data was available), otherwise findings were narratively synthesised. Eighteen studies met inclusion. Outcome measures varied widely, including diverse approaches to CBF measurement (e.g. arterial spin labelling, autoradiography, DSC-MRI), immunohistochemical measures (e.g. collagen-IV, laminin, CD31), and diverse approaches to measurement of BBB leakage (e.g. fibronectin, fibrinogen, gadolinium-based ktrans). Seven studies contributed to meta-analysis: APOE4 mice showed a consistent reduction in CBF associated with APOE4 genotype (SMD = -2.87, 95% CI: -5.14 to -0.604, df = 2.66), and a negative non-significant trend towards reduced vascular morphology expression. Narrative synthesis identified three key mechanistic pathways linking APOE4 to vascular dysfunction: (i) insulin resistance and PI3K/AKT-mTOR signalling, (ii) Cyclophilin A–NFκB–MMP9 activation, and (iii) occludin/ECM remodelling. Risk of bias assessment revealed frequent shortcomings in randomisation, blinding, and sample size justification.

**Conclusions:**

Preclinical evidence demonstrates that APOE4 drives alterations in vascular functioning primarily through involvement with pathways related to vascular metabolism, ECM remodelling and BBB leakage. However, heterogeneity in the model (e.g. age, sex, techniques), restricts direct comparability across studies. As such, standardisation or clarification of methodological approaches are necessary for rigorous assessment in the future.

**Supplementary Information:**

The online version contains supplementary material available at 10.1186/s13195-026-02018-3.

## Background

Apolipoprotein E (APOE) is a multifunctional lipid transporter involved in lipoprotein metabolism and cholesterol homeostasis [1, 2]. The E4 variant is the strongest genetic risk factor for late-onset Alzheimer’s disease (AD) [3–5] and has also been linked to other disorders, including vascular dementia, atherosclerosis, and cardiovascular disease, through mechanisms such as amyloid-β (Aβ) accumulation, neurodegeneration, and neuroinflammation [6–8]. Heterozygosity for APOE4 increases AD risk 3–4-fold, while homozygosity raises it by ~ 15-fold [9, 10]. Although APOE4 is the second most common isoform globally, its frequency varies substantially by ancestry and geographic region. Approximately 20–25% of individuals of European ancestry carry at least one E4 allele; however, frequencies are higher in certain Central African, Oceanic, and Indigenous populations and lower in Southern Europe and parts of East Asia [11]. In contrast, 40–65% of patients with Alzheimer’s disease (AD) are E4 carriers. As of 2020, nearly 50 million people worldwide were living with AD or related dementias [10, 12–14].

The mechanisms by which E4 increases dementia risk remain incompletely understood despite extensive study. Recently, several Aβ-targeting therapies have demonstrated robust biomarker outcomes but carry an elevated risk of Amyloid-Related Imaging Abnormalities (ARIA) in APOE4 carriers [15]. ARIA manifests as cerebral oedema or haemorrhage on MRI following Aβ immunotherapy and can, in severe cases, cause morbidity or death [16]. This underscores the need for a deeper understanding of the molecular and cellular mechanisms driving APOE4-related pathology to inform safer, more precise interventions.

Human studies of APOE4 pathophysiology are constrained by limited E4 homozygosity (~ 9.6% in AD patients [17] ) and the invasiveness of outcome measures. Rodent models, however, permit controlled investigation of cellular and molecular mechanisms using transgenic manipulations that mimic human disease. These models enable access to tissue-level endpoints, high reproducibility, and defined experimental conditions. Several humanised APOE mouse lines are available, some incorporating AD-related mutations in amyloid precursor protein (APP), microtubule-associated protein tau (MAPT), or familial AD (FAD) genes [18].

Beyond central nervous system alterations, APOE4 also contributes to vascular dysfunction during disease progression. A key mechanism involves blood–brain barrier (BBB) breakdown [19–22], which can be assessed through non-invasive measures such as cerebral blood flow and contrast-enhanced permeability. Additional tools—including immunohistochemistry, in vivo MRI, and behavioural testing—provide complementary insight into vascular morphology, structural integrity, and cognitive outcomes.

This review synthesises current evidence on APOE-driven vascular alterations in mouse models, focusing on genotype-related differences in BBB integrity, vascular morphology, and cerebral blood flow. Given the heterogeneity of study designs, outcomes, and ages assessed, these comparisons are crucial for building a coherent picture of APOE4-related vascular pathology and its contribution to disease progression.

## Methods

### Identification and search strategy

This review followed the PRISMA 2020 guidelines [23] and was registered in PROSPERO (CRD420251010665), and the detailed search strategy was separately registered with SearchRxiv (DOI: 10.1079/searchRxiv.2025.01124) to enhance methodological transparency and reproducibility. Literature searches were conducted in Scopus, MEDLINE, Embase, and Web of Science between 25 March and 3 April 2025. The MEDLINE search strategy was: *(Apolipoproteins E/ OR Apolipoprotein E3/ OR Apolipoprotein E4/) AND (mice/ OR mice*,* transgenic/) AND ((brain/ OR blood–brain barrier/) OR (perfusion/ OR cerebrovascular circulation/))*; full strategies for other databases are provided in Supplementary Table 1. Additional manuscripts identified by manual search were included. Unpublished data and conference abstracts were excluded.

### Study selection and data extraction

Screening and data extraction were performed using the Systematic Review Facility (SyRF) platform [24]. Two reviewers (K.L. and N.F.) independently screened titles, abstracts, and full texts, resolving disagreements by consensus with a third reviewer (A.M.).

Eligible studies:

1. Used humanised targeted replacement APOE mice (APOE3-TR, APOE4-TR) [25] or APOE-TR;5xFAD (EFAD) lines [26].
2. Provided comparative analysis between APOE3 and APOE4 homozygotes; APOE4-only studies without APOE3 controls were also accepted [27–29].
3. Assessed cerebrovascular structure or function (e.g., cerebral blood flow, vascular density, permeability, pericyte coverage).

Exclusion criteria included non-mice models, human studies, irrelevant outcomes (e.g., amyloid pathology only), non-vascular interventions, or descriptive designs. Additional criteria appear in Supplementary Table 2.

Key study characteristics (strain, age, design) were extracted by one reviewer (K.L.) and validated by another (N.F.) (Table 1). Outcome data were extracted independently and consolidated into a consensus dataset. Graphical data were digitised using WebPlotDigitizer [30]. No major discrepancies were identified.

**Table 1:** List of studies selected for review, including strain, group size, age, and sex

Study	Strain (Supplier)	Group Size	Age (s)	Sex	Ref
Nishitsuji et al., 2011 [31]	Human APOE knock-in mice generated by homologous recombination; C57BL/6 background (substrain not specified). Wild-type C57BL/6 mice purchased from SLC Inc. (Hamamatsu, Japan).	-	Primary cultures of mouse brain capillary endothelial cells (mBECs) were prepared from 3-week-old mice	Not specified	[31]
**Bell et al.**,** 2012 **[6]	TR-APOE mice generated in-house; backcrossed for ≥ 8 generations onto a C57BL/6J background (> 99.6% C57BL/6J).	*n* = 5 /group	4–9 months	Not specified	[6]
**Alata et al.**,** 2015 **[32]	APOE (E2, E3, E4) targeted replacement mice on a C57BL/6 background (substrain not specified); Taconic Transgenic Models (Hudson, NY, USA).	*n* = 5–6/group	12 months	Male, Female	[32]
**Thomas et al.**,** 2017 **[33]	EFAD (5xFAD × APOE-TR), mixed C57BL/6 × SJL background (substrain not specified); 5xFAD Tg6799 (Vassar lab); APOE-TR from Taconic; breeding at Taconic Laboratories.	*n* = 6–8/group	6-8.5 months	Male, Female	[33]
Lin et al., 2017 [34]	Wild-type (C57BL/6, substrain not specified) and APOE4 transgenic mice (human APOE4 under GFAP promoter; murine *Apoe* null), Jackson Laboratory (Bar Harbor, ME, USA).	*n* = 6/group	1–7 months	Female	[34]
**Marottoli et al.**,** 2017** [35]	EFAD (5xFAD × APOE-TR) mice expressing human APOE3 or APOE4; mixed C57BL/6 × SJL background (substrain not specified). Generated by crossing APOE-TR (C57BL/6) with 5xFAD (C57BL/6 × SJL) mice; colony founders provided by Dr. M. J. LaDu.	*n* = 8/group	4–6 months	Male	[35]
**Koizumi et al.,** 2018 [7]	ApoE3-TR and ApoE4-TR mice on a C57BL/6 background (substrain not specified); C57BL/6 wild-type controls (origin not reported).	*n* = 5/group	3–4 months	Male	[7]
Johnson et al., 2019 [36]	Homozygous human APOE3-TR and APOE4-TR mice (Sullivan et al., 1997); generated in 129 ES cells and crossed with C57BL/6J mice (background not further specified in study).	Cerebral Blood Volume measure: *n* = 7–8/group	15 months	Female	[36]
**Yamazaki et al.**,** 2020** [37]	ApoE-targeted replacement (APOE3-TR, APOE4-TR; mouse Apoe promoter-driven) and Apoe-knockout mice on a C57BL/6 background (substrain not specified); Taconic (Hudson, NY, USA).	*n* = 4/group	22 months	Male, Females	[37]
Ringland et al., 2020 [38]	APOE-TR (E2/E3/E4) mice, C57BL/6 background (substrain not stated); Taconic Biosciences (Rensselaer, NY, USA). EFAD (5xFAD × APOE-TR), mixed C57BL/6 × SJL background (substrain not specified); provided by Dr. M. J. LaDu.	*n* = 4 for each genotype	6 months	Female	[38]
Lin et al., 2020 [39]	EFAD (5xFAD × APOE-TR; E4FAD and E3FAD lines), mixed C57BL/6 × SJL background (substrain not specified); breeders originally obtained from Dr. M. J. LaDu.	*n* = 8/group	2 months	Male, Female	[39]
**Montagne et al.**,** 2021** [20]	E3FAD/E4FAD (5xFAD × APOE-TR), mixed C57BL/6 × SJL background (substrain not stated); APOE3/APOE4 knock-in controls on C57BL/6 background (substrain not specified); breeders provided by Dr. M. J. LaDu.	IHC: *n* = 5–6/group BBB: *n* = 6–8/ group CBF: *n* = 12–14/group	18–24 months	Male, Female	[20]
Rhea et al., 2021 [25]	Homozygous human APOE3-TR and APOE4-TR mice; genetic background not specified; bred at Oregon Health & Sciences University (OHSU).	*n* = 10/group	15 months	Male, Female	[25]
Barisano et al., 2022 [40]	APOE3 and APOE4 KIflox/flox (E3F/E4F); Maintained on C57BL/6J background; targeting performed in Taconic C57BL/6 N ES cells.	*n* = 8/group	2–3, 4–6, and 9–12 months	Male, Female	[40]
Jackson et al., 2022 [41]	Conditional humanised APOE knock-in (APOE2/3/4 KI) mice on C57BL/6 background (substrain not specified); Apoe knockout and ALDH1L1-Cre lines from The Jackson Laboratory; huAPOE KI lines per Taconic protocols.	*n* = 6/group	8-8.5 months	Not specified	[41]
Yanckello et al., 2022 [42]	E3FAD/E4FAD (5xFAD × APOE-TR), mixed C57BL/6 × SJL background (substrain not stated); genotyped by Transnetyx Inc. (Cordova, TN, USA).	*n* = 8/group; (male: female = 1:1)	7 months	Male, Female	[42]
Onos et al., 2024 [43]	B6J.APOE3 KI (hAPOEε3; JAX#029018) and B6J.APOE4 KI (hAPOEε4; JAX#027894) mice; C57BL/6J background; Jackson Laboratory (Bar Harbor, ME, USA). hAPOEε3/ε4 generated by intercrossing these strains.	*n* = 2–5/group	4–12 months	Male, Female	[43]
Bhattarai et al., 2025 [44]	Human APOE replacement mice (Sullivan et al., 1997) [45]; generated in 129 ES cells and crossed with C57BL/6J mice (background not fully specified in experimental generation). APP NL-G-F knock-in mice; background not explicitly stated (C57BL/6J non-transgenic controls used).	*N* = 6 (50% female) for every experiment	18–22 months of age	Male, Female	[44]

Studies included in synthesis are in bold

### Risk of Bias (RoB) and quality assessment

Study quality and risk of bias were evaluated using established preclinical tools: the CAMARADES checklist [46] for overall study quality and the SYRCLE Risk of Bias tool [47] for individual bias domains. Assessments were conducted by one reviewer (K.L.) and cross-checked on a subset of studies by a second reviewer (N.F.) to ensure consistency.

### Synthesis methods

Meta-analyses assessed the impact of APOE genotype on cerebral blood flow (CBF) and vascular morphology in humanised APOE knock-in or targeted replacement (APOE-KI/TR) mice using RStudio (version 2024.12.1). Key study characteristics—experimental design, outcomes, age, and region analysed—were tabulated, and studies grouped by outcome domain (CBF or vascular morphology). Only those providing sufficient quantitative data (mean, SEM, and sample size) for APOE3 versus APOE4 comparisons were included in meta-analyses; others were summarised narratively. SEMs were converted to standard deviations using:

$$SD=SEM\times\surd{n}$$

Multiple studies compared different ages and brain subregions, resulting in several layers of analysis within a single study [6, 7, 20, 34, 48]. Studies which contributed multiple subgroup comparisons (e.g., data from different brain regions or age groups) were treated as separate data points and analysed independently. Each subgroup was assigned an alphabetical suffix (e.g., a, b, c) and specific comparisons were detailed in the captions of forest plots.

Given expected heterogeneity in design, imaging methods, and antibodies, all quantitative syntheses employed random-effects models. When studies contributed multiple within-study comparisons, a hierarchical random-effects model using robust variance estimation (RVE) accounted for non-independence of effect sizes. Between-group differences (APOE3 vs. APOE4) were expressed as standardised mean differences (SMDs) with 95% confidence intervals (CIs). Where multiple measures were reported for the same group, pooled values were calculated following CAMARADES guidelines [49].

As traditional heterogeneity metrics (Cochran’s Q, I²) are not available with RVE, τ² was reported to represent between-study variance. For analyses with a single effect size per study, standard random-effects models (metafor package, R) were used, providing SMDs, 95% CIs, and heterogeneity indices (τ², I², Q).

Formal meta-analysis was not undertaken for outcomes with fewer than three studies. In these cases, results were summarised narratively, and consistency across subgroups and study characteristics was assessed qualitatively.

## Results

### Literature retrieval

The database search identified 1,493 records, with three additional studies found through manual searching. After removal of five duplicates, 1,488 titles and abstracts were screened. Of these, 1,438 were excluded: 711 were irrelevant; 17 involved the wrong population (e.g., human studies [50] ); 109 used inappropriate designs (e.g., amyloid clearance [38, 51], gene editing, nanomedicine); 384 employed unsuitable models (e.g., ApoE−/−, APP, or Lrp1-KO mice [52] ); 79 used unrelated interventions (e.g., traumatic brain injury, artery occlusion, chronic inflammation [53] ); and 138 reported unrelated outcomes (e.g., amyloid burden, behaviour, transcriptomics).

**Fig. 1:**
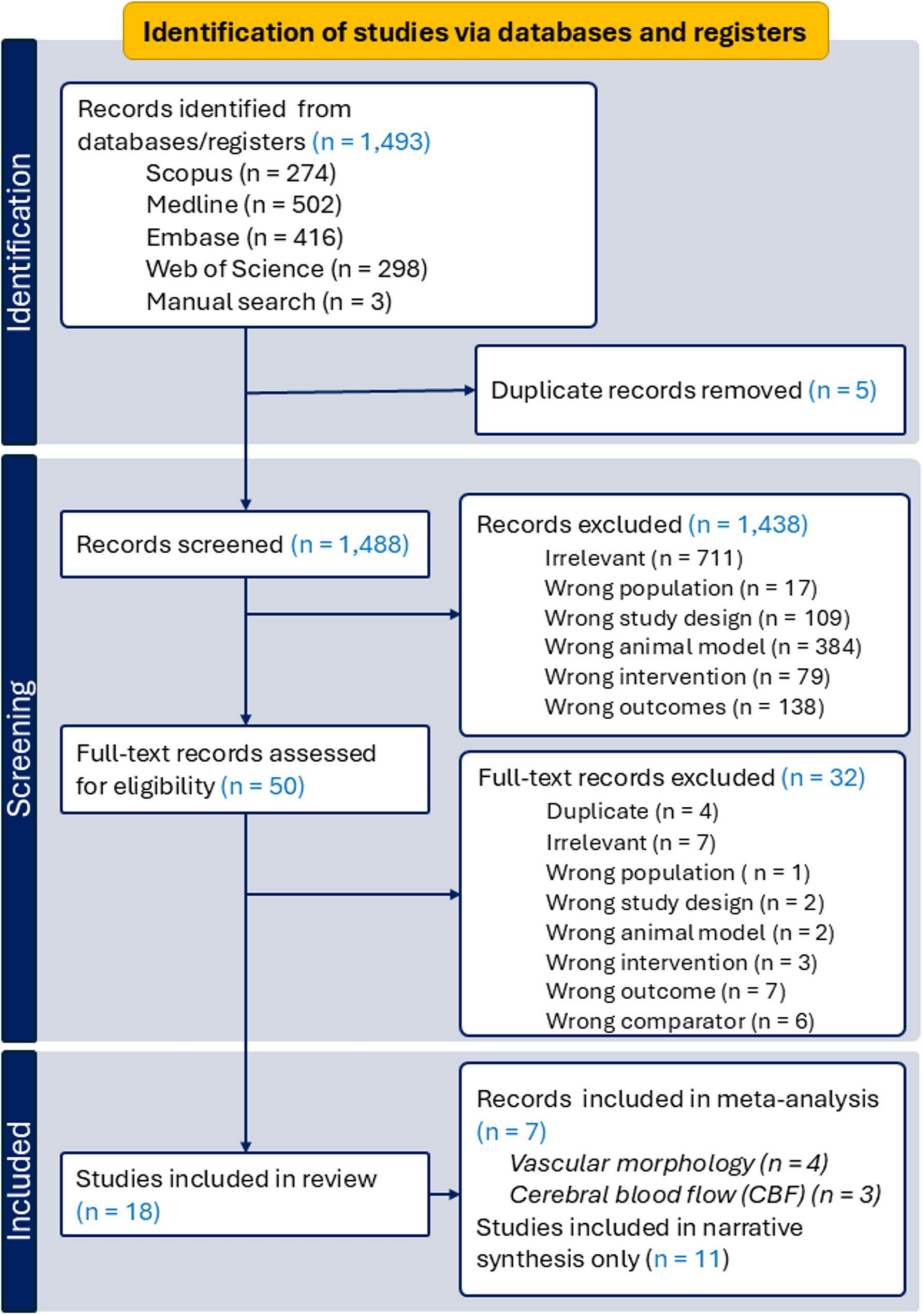
Flow diagram of study selection process following PRISMA 2020 guidelines

Fifty studies underwent full-text review, and 18 met inclusion criteria for the systematic review (Fig. 1). Of these, seven were eligible for meta-analysis: six reported vascular morphology (four unique, two overlapping with CBF outcomes), and three examined genotype-dependent differences in cerebral blood flow. The remaining 11 were synthesised narratively. Study characteristics and exclusion details are provided in Supplementary Table 3.

### Study characteristics

Characteristics of the 18 included studies are summarised in Table 1. All used transgenic murine models expressing human APOE alleles, with variation in strain, background, supplier, group size, age, and sex. Nine studies employed C57BL/6 background mice, while others used outbred strains, EFAD lines [20, 33, 35, 39], or did not specify background. Mouse ages ranged from 2 to 24 months, encompassing early life (2–4 months), midlife (6–12 months), and later life (up to 24 months).

Methodologies also varied, including assessments of BBB permeability (e.g., gadolinium-based Ktrans, fibrinogen, fibronectin), vascular morphology, and cerebral blood flow. Detailed PICO (Population, Intervention, Comparator, Outcome) information for each study is provided in Table 2.

**Table 2:** PICO elements (Population, Intervention, Comparator, Outcome) across studies included in the quantitative synthesis

Study	Comparator	Intervention	Outcome Domain	Outcome Measure	Time Points	Brain Regions
Bell et al., 2012 [6]	APOE3 vs. APOE4	BBB	Pericyte Coverage	CD13 + IHC (% pericyte coverage)	2 weeks, 9 months	Cortex, Hippocampus
		CBF	CBF	14 C-iodoantipyrine autoradiograms	2 weeks, 9 months	Sensorimotor, Ectorhinal Cortex, Hippocampus, Caudate Putamen, Corpus Callosum, Thalamus
Alata et al., 2015 [32]	APOE3 vs. APOE4	BBB	Basement Membrane Integrity	Collagen-IV IHC (% area occupied)	12 months	Cortex
Thomas et al., 2017 [33]	APOE3 vs. APOE4 (E3FAD vs. E4FAD)	BBB	BBB Leakage	Fibrinogen IHC (% area occupied)	22 months	Cortex
Marotolli et al., 2017 [35]	APOE3 vs. APOE4 (E3FAD vs. E4FAD)	BBB	BBB Leakage	Fibrinogen IHC (% area occupied)	4–6 months	Cortex
Koizumi et al., 2018 [7]	APOE3 vs. APOE4	BBB	Basement Membrane Integrity	Laminin IHC (% area occupied)	3–4 months	Cortex
		CBF	CBF	ASL-MRI	3–4 months	Cortex, Caudate Nucleus
Yamazaki et al., 2020 [37]	APOE3 vs. APOE4	BBB	Basement Membrane Integrity	Collagen-IV immunofluorescence (relative intensity)	22 months	Cortex
Montagne et al., 2021 [20]	APOE3 vs. APOE4	BBB	Pericyte Coverage	CD13 + IHC (% coverage)	18–24 months	Cortex, Hippocampus
			BBB Leakage	Fibrinogen IHC (integrated density ×10³)	18–24 months	Cortex, Hippocampus
				DCE-MRI Ktrans (×10⁻³ min⁻¹)	18–24 months	Cortex, Hippocampus
		CBF	CBF	ASL-MRI	18–24 months	Cortex, Hippocampus

*BBB Integrity* refers to structural vascular markers (e.g., collagen-IV, laminin, Glut1), whereas *BBB Leakage* refers to extravasation or permeability measures (e.g., fibrinogen, IgG, Ktrans, tracer assays). All studies provided mean ± SEM and sample size data sufficient for effect size estimation

### Quality assessment and risk of bias

Study quality and reporting were evaluated using the CAMARADES checklist and SYRCLE Risk of Bias tool across all 18 studies. The mean CAMARADES score was 7.2/10 (72%), ranging from 4 to 9.5 (Table 3). All studies used appropriate transgenic mouse models and adhered to animal welfare regulations; all but one (Bhattarai et al., 2025, *bioRxiv*) were peer-reviewed. Owing to the transgenic design, all studies met the criterion for blinded induction. However, several domains showed incomplete reporting, and only four studies explicitly described sample size calculations.

**Table 3:** CAMARADES checklist for study quality

Study	1	2	3	4	5	6	7	8	9	10	Score
Nishitsuji et al., 2011 [31]	x			x			x		x		**4**
***Bell et al., 2012*** [6]	x		x	x	x	x	x		x	x	**8**
***Alata et al., 2015*** [32]	x	x		x		*	x		x	x	**6.5**
***Thomas et al., 2017*** [33]	x			x	x	*	x		x	x	**6.5**
Lin et al., 2017 [34]	x	x		x	x	x	x		x	x	**8**
***Marottoli et al., 2017*** [35]	x		x	x	x		x		x	x	**7**
***Koizumi et al., 2018*** [7]	x	x		x	x	x	x		x	x	**8**
Johnson et al., 2019 [36]	x	x		x		*	x		x	x	**6.5**
***Yamazaki et al., 2020*** [37]	x			x		*	x		x	x	**5.5**
Ringland et al., 2020 [38]	x	x		*			x		x	x	**5.5**
Lin et al., 2020 [39]	x	x	x	x	x	*	x	x	x	x	**9.5**
***Montagne et al., 2021*** [20]	x	x	x	x	x	x	x		x	x	**9**
Yamazaki et al., 2021 [69]	x	x		x		x	x		x	x	**7**
Rhea et al., 2021 [25]	x		x	x		x	x	x	x	x	**8**
Barisano et al., 2022 [40]	x	x	x	x	x	x	x		x	x	**9**
Jackson et al., 2022 [41]	x	x		x		x	x		x	x	**7**
Yanckello et al., 2022 [42]	x		x	x			x	x	x	x	**7**
Bonnar et al., 2023 [54]	x	x		x	*	x	x	x	x	x	**8.5**
Onos et al., 2024 [43]	x	x	x	x	x	x	x		x	x	**9**
Anderle et al., 2025 [55]	x	x		x		x	x		x	x	**7**
Bhattarai et al., 2025 [44]		*	x	x		x	x		x		**5.5**

Each study was assessed against the following criteria: (1) publication in a peer-reviewed journal; (2) statement of temperature control; (3) random allocation to treatment or control; (4) blinded induction of the model; (5) blinded assessment of outcome; (6) use of anaesthetic without significant intrinsic neuroprotective activity; (7) use of an appropriate animal model; (8) sample size calculation; (9) compliance with animal welfare regulations; (10) statement of potential conflicts of interest. “Ref” indicates references. An “x” denotes fulfilment of the criterion (score = 1); a blank cell indicates non-fulfilment. An asterisk (*) represents partial fulfilment (score = 0.5). See supplementary material for additional details. Studies in bold italics were included in the meta-analysis. Studies included in the quantitative synthesis are indicated in bold

Several domains exhibited unclear risk of bias, particularly performance bias, selection bias, and detection bias, due to insufficient reporting (Supplementary Table 4). Randomised housing was poorly reported in most studies. In contrast, attrition bias, reporting bias, and other sources of bias (e.g., conflict of interest declarations) were most consistently rated as low risk. Only Onos et al., (2024) was rated low risk across all SYRCLE domains.

### Study outcomes

Included studies assessed diverse yet overlapping aspects of blood–brain barrier breakdown (BBB-b). Five studies reported cerebral blood flow changes [6, 7, 20, 34, 39], and three examined tight junction integrity [6, 31, 32]. Ten studies identified cerebrovascular morphological differences by APOE genotype, including vessel density, pericyte coverage, vessel diameter, and vascular volume [6, 7, 20, 25, 32–35, 37, 41].

Mechanistic investigations varied: one study conducted multi-omic profiling (transcriptomic, lipidomic, proteomic) [40], and two evaluated neurovascular coupling in the visual cortex [54, 55]. Three studies explored Cyclophilin-A–NFκB–MMP9 signalling [6, 34, 38], while seven addressed pathways involving insulin resistance, metabolism, and mTOR signalling [25, 34, 36, 39, 43, 48, 56]. Additional studies examined EGF-related resilience [33], peripheral inflammation [35], and glial contributions to BBB-b [7, 37, 41, 44].

Given the limited number of studies per outcome domain, formal assessments of reporting bias (e.g., funnel plot asymmetry, Egger’s test) were not performed.

### APOE4 contributions to BBB destabilisation and meta-analysis

#### Cerebral blood flow

**Fig. 2:**
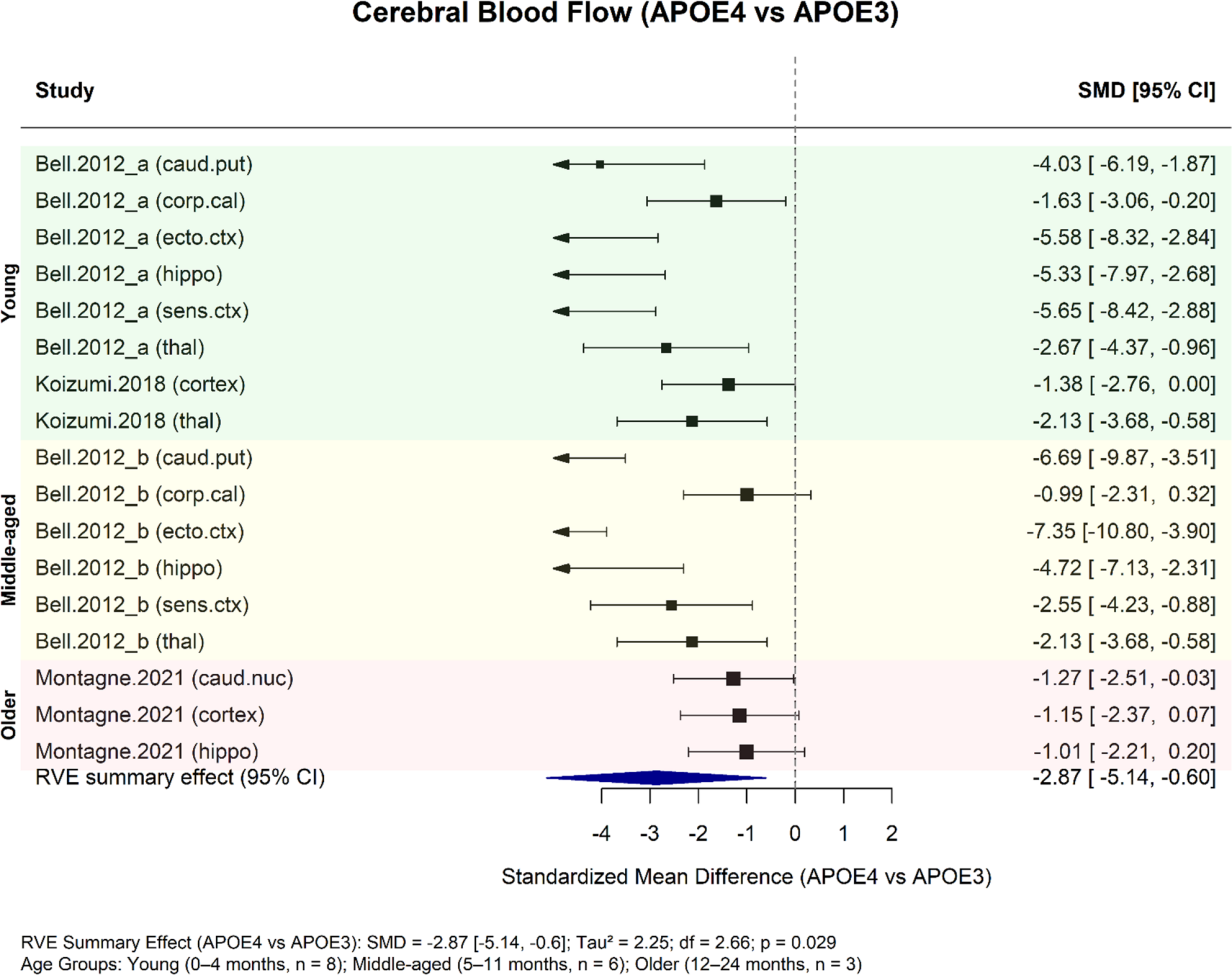
Meta-analysis of cerebral blood flow differences between APOE genotypes by age and brain subregion, using robust variance estimation (RVE). Standardised mean differences (SMDs) comparing APOE4 vs. APOE3 were derived from outcome comparisons of brain region and age across three studies. RVE was used to account for within-study clustering, with four distinct clusters (Montagne et al., 2021 [20]; Koizumi et al., 2018 [7]; and Bell et al.,2012 [6], which was divided into clusters “a” and “b” representing two age groups). The RVE summary effect is shown as the dark blue polygon. Between-study heterogeneity was moderate-to-substantial (τ2 = 2.25). Samples sizes were not adjusted for repeated comparisons within studies due to small group sizes, and degrees of freedom were limited (2.66)

Three studies assessed APOE-dependent differences in cerebral blood flow (CBF) across distinct age groups and brain regions, using dynamic susceptibility-contrast MRI (DSC-MRI) [20], arterial spin labelling MRI (ASL-MRI) [7], or autoradiography with radiolabelled tracers [6]. Despite methodological variation, RVE meta-analysis indicated a consistent reduction in CBF in APOE4 mice relative to APOE3 (SMD = − 2.87, 95% CI − 5.14 to − 0.60, df = 2.66; Fig. 2). Between-study heterogeneity was moderate to substantial (τ² = 2.25), reflecting differences in imaging modality.

All three studies were of moderate-to-high quality (mean CAMARADES score 8.3/10). However, risk of bias may have arisen from incomplete reporting of sample size calculations and uncertainty in performance bias domains (e.g., randomised housing). Overall, 10–60% of SYRCLE items were rated as unclear risk. 

#### Vascular morphology

Six studies investigated APOE genotype–dependent differences in vascular morphology using immunohistochemical markers. Two assessed laminin coverage as cortical percent area in 5- and 8-month-old EFAD mice [33, 35]. Three evaluated collagen-IV expression as a marker of vessel density—quantified as hippocampal capillary area in 9- and 12-month-old mice [6, 35] or cortical signal intensity in 22-month-old mice [37]. One measured neocortical microvessel density via CD31 immunostaining in 9-month-old mice [7].

**Fig. 3:**
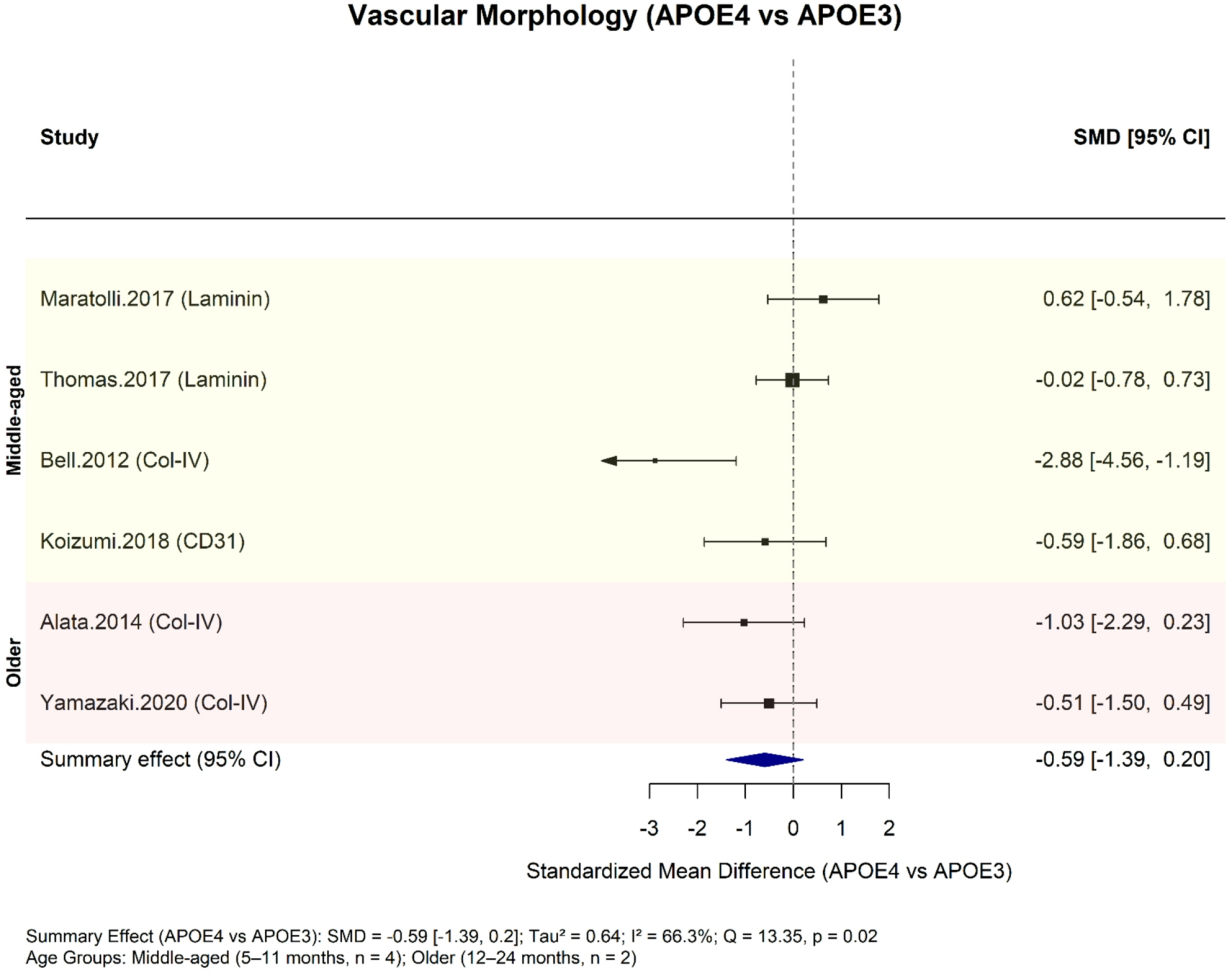
Meta-analysis of vascular morphological differences between APOE genotypes using a random-effects model. Standardised mean differences (SMDs) comparing APOE4 mice vs. APOE3 mice were calculated across five studies assessing vascular morphology using IHC markers (laminin, collagen-IV, CD31). The summary effect is shown as the dark blue polygon. Between-study heterogeneity was moderate (τ2 = 0.64)

A random-effects meta-analysis (k = 6) showed a negative but non-significant trend toward reduced vascular morphology markers in APOE4 versus APOE3 mice (SMD = − 0.59; 95% CI − 1.39 to 0.20; *p* = 0.14; Fig. 3). Heterogeneity was moderate to substantial (τ² = 0.64; I² = 66.3%; Q = 13.35, *p* = 0.020).

Included studies had a mean CAMARADES score of 6.9/10. Risk of bias reflected incomplete reporting of temperature control, randomisation, and sample size calculation. According to SYRCLE, 30–60% of domains were rated unclear, primarily for selection and performance bias (Supplementary Table 4).

### Narrative summary of APOE4-associated mechanistic pathways

Remaining studies that could not be quantitatively synthesised were analysed narratively to provide complementary mechanistic insights (Table 4).

**Table 4:** Summary of key mechanistic axes implicated in APOE4-related blood–brain barrier (BBB) dysfunction

Mechanistic Axis	Studies	Key Pathways/Markers	Common Findings
Insulin/mTOR signalling	- Lin 2020 - Johnson 2019 - Rhea 2020 - Onos 2024	Insulin receptor availability, mTOR, decreased glucose uptake	APOE4 linked to metabolic dysregulation
Cyclophilin A – NFκB – MMP9	- Lin 2017 - **Bell 2012** - Ringland 2020 - Jackson 2022	MMP9, CypA, NFκB activation	Vascular breakdown mediated by pericyte-linked inflammation.
Occludin & ECM	- Nishitsuji 2011 - **Yamazaki 2020 ** - **Thomas 2017** - Bhattarai 2025 - Yanckello 2022 - Barisano 2022	TEER, Tight junction (claudin-5, occludin), Fibrinogen, Fibronectin, Collagen IV	Increased permeability and ECM disruption in APOE4

Studies included in the quantitative synthesis are indicated in bold

#### Insulin/mTOR signalling

Several studies have investigated the role of insulin resistance in brain metabolism and endothelial dysfunction in APOE4 mice, often focusing on the mammalian target of rapamycin (mTOR) signalling pathway.

Johnson et al. [36] reported that high-fat diet (HFD)-induced insulin resistance was associated with impaired cognition, reduced cerebral blood volume (CBV), and decreased glucose uptake, with more pronounced effects in 15-month-old APOE4 than APOE3 mice. Administration of an acute exogenous glucose bolus produced a paradoxical improvement in CBV and cognition for APOE4 mice only, demonstrating a significant genotype effect (*p* = 0.028).

Onos et al. [43] further confirmed genotype-linked alterations in glucose metabolism. Longitudinal FDG-PET imaging revealed widespread reductions in glucose uptake in APOE4 mice across multiple brain regions, with complex age- and sex-specific dynamics. Fluctuating uptake patterns suggested a Type 1 neurovascular uncoupling process - where decreased glucose uptake co-occurs with increased perfusion at 8 and 12 months.

Rhea et al. [25] examined insulin pharmacokinetics in 15-month-old APOE-TR mice, identifying genotype- and sex-specific differences in vascular insulin binding. APOE4 females exhibited higher amounts of reversible binding of ^125^I-Insulin in the frontal cortex, than APOE3 females, proposed to reflect differences in insulin receptor availability or binding site accessibility on the luminal surface of brain endothelial cells. However, downstream dynamics regarding insulin transport across the BBB remained unclear.

Lin et al. [39] further linked APOE4-associated vascular dysfunction to overactivation of mTOR. In 7-month-old E4FAD mice, mTOR hyperactivity was associated with reduced P-glycoprotein (P-gp) transport at the BBB (*p* < 0.001), impaired CBF, disrupted lipid metabolism, and elevated free fatty acids (FFAs). Sixteen weeks of rapamycin treatment restored BBB function and lipid homeostasis in APOE4 mice. Interestingly, rapamycin administration in APOE3 mice slowed glucose-associated metabolism – reducing glycolytic and TCA cycle intermediates – whereas *APOE4* metabolism was normalised. This effect was suggested to mirror caloric restriction-like outcomes, where mTOR inhibition in relatively healthy animals reduces glucose uptake.

Together, these findings support a model in which the APOE4 genotype is associated with dysregulated brain insulin signalling, reduced cerebral glucose uptake, and a paradoxical relationship with mTOR activation processes further modified by diet, age, and sex. This raises the possibility that APOE4 may confer a state of chronic endothelial metabolic vulnerability, which becomes increasingly destabilised in the presence of modifiable risk factors. Within this framework, mTOR overactivation appears to function maladaptively in APOE4 mice, contributing to impaired insulin signalling and vascular function.

#### Cyclophilin A – NFκB – MMP9

Four studies demonstrate that the APOE4 genotype contributes to blood-brain barrier (BBB) breakdown via activation of the Cyclophilin A (CypA) – Nuclear Factor kappa B (NFkB) – matrix metalloproteinase 9 (MMP9) pathway.

Bell et al. [6] reported a 5-6-fold increase in CypA levels within the cerebral microvessels of 6-month-old APOE4 mice under the GFAP promoter, primarily localised to pericytes. Further experiments demonstrated that this increase drives NFkB and MMP9 expression, resulting in pericyte-mediated ECM remodelling. Pharmacological inhibition of CypA with cyclosporine A, an immunosuppressant, attenuated these effects on BBB integrity.

Lin et al. [34] corroborated CypA’s role in APOE4-related vascular dysfunction. Here, treatment with rapamycin in 12-month-old APOE4 mice under the GFAP promoter normalised CypA and NFkB levels in cortical parenchyma and isolated brain microvessels, improved BBB integrity, and restored CBF (ASL-MRI). Although total brain CypA levels were not significantly different between genotypes, APOE4 control mice showed reduced parenchymal and elevated vascular CypA levels, indicating regional heterogeneity in CypA expression.

Ringland et al. [38] found impaired regulation of MMP9 in 6-month-old E4FAD mice, reporting a 56% increase in MMP9 immunoreactivity in cortical endothelial cells compared to E3FAD. At 70 weeks, E4FAD mice continued to show elevated MMP9 levels relative to E3FAD mice (*p* = 0.0533), suggesting chronic dysregulation.

In vivo experiments by Jackson et al. (2022) confirmed BBB disruption in APOE4 mice by 9-months-old. Using fluorescein-labelled 40 kDa dextran in tracer extravasation studies, increased permeability was observed. Quantitative PCR revealed a ~ 30% increase in MMP9 transcript levels in *APOE4* mice, accompanied by elevated MMP9 protein levels. Transmission electron microscopy showed irregular gaps in tight junction membranes, suggesting focal openings rather than a global loss of structure. Additionally, measurement of astrocytic end-foot coverage around vessels demonstrated a significant reduction.

Together, these studies implicate APOE4 in activating a proinflammatory and extracellular matrix remodelling cascade involving pericyte-endothelial interactions. This pathway contributes to BBB breakdown, including localised tight junction dysregulation, detectable as early as 6 months of age. The convergence of elevated CypA and NFkB signalling with sustained MMP9 activity supports early pericyte-mediated involvement in enzymatic ECM remodelling rather than passive vascular decline. The regional heterogeneity of CypA expression and the focal nature of tight junction openings further suggest that degeneration in APOE4 mice may not manifest as a uniform barrier collapse, but instead as a spatially selective and progressively destabilising vascular dysfunction.

#### Occludin & ECM

The APOE4 genotype has been strongly associated with compromised BBB integrity, driven by both structural and functional disruptions of endothelial and perivascular components. Several studies highlight specific markers of BBB dysregulation, including tight junction protein, fibrinogen, fibronectin and extracellular matrix components.

Nishitsuji et al. [31] employed an in vitro BBB model incorporating primary astrocytes from APOE3- or APOE4-knock-in mice and found significantly reduced trans endothelial electrical resistance (TEER) in the *APOE4* condition. While findings revealed no change in total tight junction protein levels, phosphorylation of occludin at threonine (Thr) residues – required for tight junction assembly – was significantly reduced. This was accompanied by diminished protein kinase C (PKC)η activation, which has been shown to regulate the phosphorylation of occludin [57]. These findings are consistent with impaired LRP1–PKCη–occludin signalling, ultimately compromising tight junction integrity.

Yanckello et al. [42] further examined tight junction protein expression in brain capillaries isolated from 7-month-old E3FAD and E4FAD mice, with or without dietary inulin (a prebiotic fibre). While inulin did not significantly alter occludin protein levels, Claudin-1 expression differed at baseline, with E4FAD control mice exhibiting elevated levels (*p* = 0.0012).

Yamazaki et al. [37] provided further evidence of APOE genotype-dependent BBB permeability using both in vitro and in vivo approaches. TEER was significantly lower in BBB models containing *APOE4*-expressing pericytes, despite no significant differences in tight junction protein levels. However, collagen-IV expression was significantly reduced in APOE4-pericyte models. Immunofluorescence confirmed diminished collagen-IV in 8-month-old APOE4 mice. ELISA assays revealed increased cortical IgG and fibrinogen, supporting a role for extracellular matrix impairment in BBB permeability and an inverse relationship between extravasating markers and basement membrane structural integrity.

Thomas et al. [33] identified that 8-month-old female E4FAD mice exhibited ~ 65% higher cortical fibrinogen levels and increased sodium fluorescein (NaFl) leakage compared to other groups, suggesting a sex-specific vulnerability of the BBB. Treatment with epidermal growth factor (EGF) attenuated fibrinogen extravasation by about 40%.

Barisano et al. [40] used dynamic contrast-enhanced MRI and histological analysis to investigate BBB integrity in APOE-targeted replacement mice. Their findings revealed progressive BBB leakage in APOE4 mice, evidenced by pericapillary fibrinogen accumulation. Tissue analysis also showed a progressive loss of pericyte coverage (CD13^+^), which strongly inversely correlated with fibrinogen deposition, linking structural loss to BBB leakiness. Transcriptomic analysis of endothelial cells revealed upregulation of genes involved in cell adhesion (e.g., cadherins, contactins, catenins), interpreted as a possible compensatory response to vascular compromise.

Finally, Bhattarai et al. [44] reported significantly elevated fibronectin accumulation in APOE4 mice - a hallmark of extracellular matrix remodelling - without changes in the CD31 endothelial marker, further demonstrating enhanced susceptibility to BBB permeability in the APOE4 genotype.

Altogether, these studies emphasise the relationship between increased BBB permeability and reduced vascular stability in the APOE4 genotype. Collectively, the findings point toward defects in tight junction regulation, particularly altered occludin phosphorylation, alongside reduced endothelial electrical resistance. Importantly, several models demonstrate that APOE4 expression in perivascular cells, including pericytes and astrocytes, can influence BBB structure and function beyond the endothelium itself. The consistent elevation of extravasation markers such as fibrinogen, in the absence of widespread vascular loss, suggests that APOE4-associated barrier dysfunction reflects impaired cell adhesion, perivascular involvement and junctional stability rather than overt vascular degeneration.

**Fig. 4:**
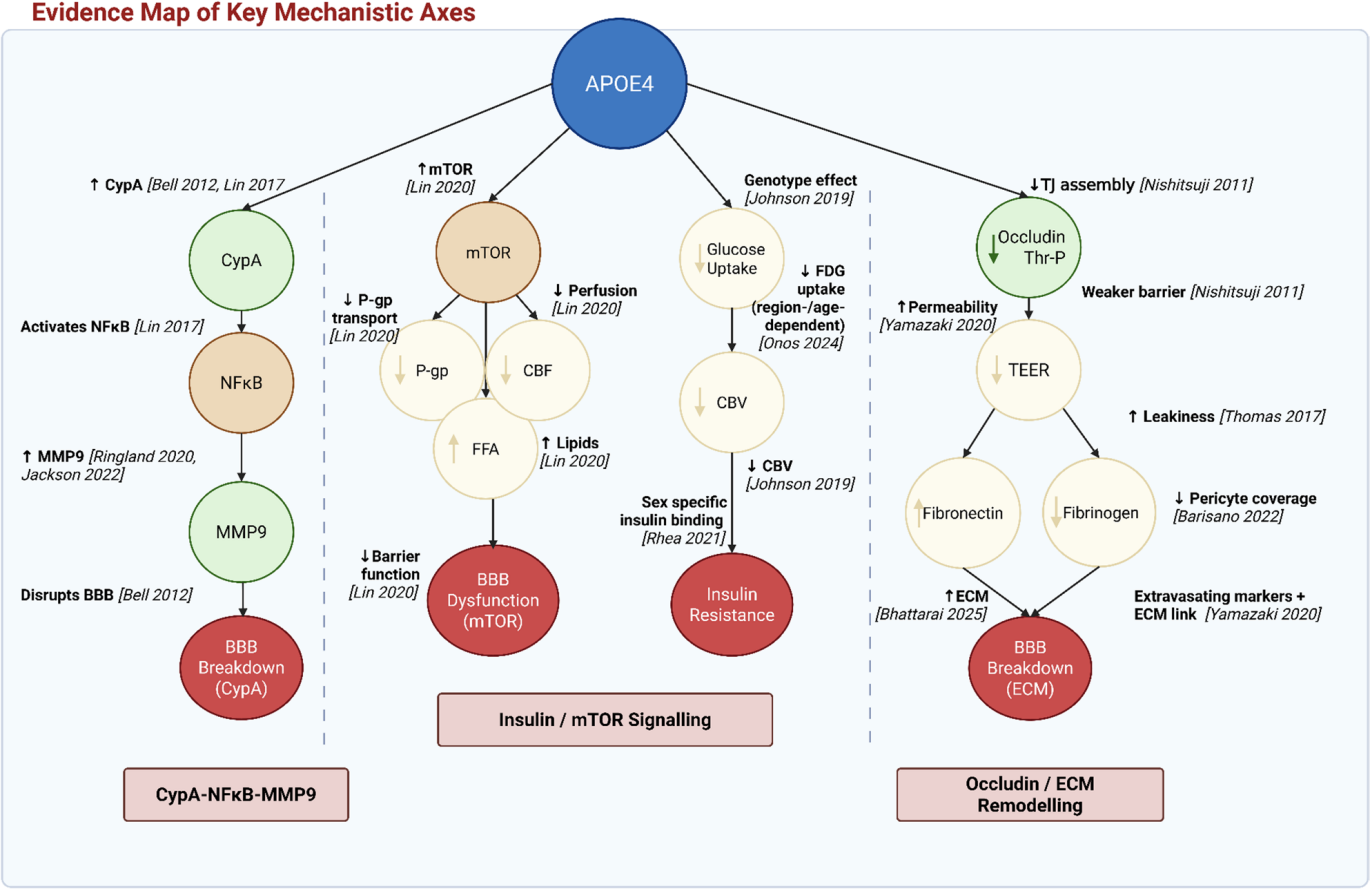
Evidence mapping schematic of studies included in the narrative synthesis. This figure illustrates three principal pathways reported in the literature: (i) CypA-NFκB-MMP9 signalling, (ii) insulin/PI3K-mTOR signalling, and (iii) occludin and extracellular matrix (ECM) integrity. Nodes are annotated with studies contributing evidence. Blue = APOE4, green = intermediate mediators, yellow = molecular/protein markers, red = outcomes. Abbreviations: CypA, cyclophilin A; NFκB, nuclear factor kappa B; MMP9, matrix metalloproteinase 9; mTOR, mammalian target of rapamycin; P-gp, P-glycoprotein; FFA, free fatty acids; CBF, cerebral blood flow; CBV, cerebral blood volume; TEER, trans-endothelial electrical resistance; FN1, fibronectin. Created in BioRender. Laing, K. [58]

Together, these three mechanistic axes provide converging evidence of progressive BBB destabilisation in APOE4 contexts (Fig. 4). This disruption is characterised by increased permeability – evidenced by tracer extravasation, blood product accumulation, and focal tight junction widening – and is accompanied by sustained inflammatory signalling, likely driven by ongoing extracellular matrix remodelling and perivascular dysfunction.

## Discussion and future perspectives

This systematic review examined associations between the Apolipoprotein E4 (APOE4) genotype and BBB integrity in humanised APOE-target replacement (TR) and knock-in (KI) mouse models. Recognising that methodological quality and bias assessment are critical for translational validity, this review began by evaluating the quality of included studies before synthesising trends in BBB structure, function, and molecular mechanisms.

Using the CAMARADES checklist and SYRCLE Risk of Bias (RoB) tool [47], we assessed 22 studies that met eligibility criteria for reporting quality. The average CAMARADES score was 7.2/10 (72%), suggesting overall satisfactory experimental design; however, several areas were consistently underreported, particularly random allocation to treatment, blinding of outcome assessment, and transparent reporting of sample size calculation. These items are often assumed or implied within the field, but lack of - or insufficient - reporting of these critical design elements could compromise experimental validity or increase the risk of bias in outcomes. This concern was further underscored by the SYRCLE RoB assessment, which identified high rates of unclear reporting in selection and performance bias domains (e.g., allocation concealment, randomised housing, and caregiver blinding), highlighting the need for more rigorous and standardised reporting practices in preclinical BBB research.

To assess genotype-related differences, meta-analyses were performed examining CBF and vascular morphology in APOE4 mice vs. APOE3 mice. Despite heterogeneity in imaging modalities (DSC- and ASL-MRI, and ^14^C-autoradiography), studies consistently reported reduced CBF in APOE4 compared with other APOE genotypes across a broad age range (0–24 months).

Analysis of vascular morphology, evaluating markers of endothelial and basement membrane integrity (laminin, collagen-IV, CD31), revealed a less straightforward trend with middle-aged mice (5–11 months) demonstrating higher vascular expression than older mice (12–24 months). Although the standard mean difference for APOE4 relative to APOE3 was not statistically significant, this pattern is notable and aligns with broader mechanistic observations, including upregulation of key components within the CypA-NFkB-MMP9 axis and evidence from the occludin/ECM remodelling pathway.

Studies within the CypA-NFkB-MMP9 axis consistently reported elevated levels of CypA, NFkB, and MMP9 – key mediators of inflammatory signalling and ECM remodelling. While MMP9 can cleave glycoproteins such as laminin, it demonstrates higher binding affinity and substrate specificity for collagens, particularly collagen-IV [59, 60]. Notably, collagen-IV downregulation was observed in APOE4 models within the occludin/ECM pathway. Jackson et al. (2022) reported elevated MMP9 transcripts in 9-month-old mice, whereas Yamazaki et al. [37] observed collagen-IV reduction in 8-month-old mice, supporting a temporal and mechanistic link between enzymatic ECM remodelling and compromised basement integrity. Together, these findings are consistent with MMP9-mediated destabilisation of structural barrier components in APOE4 contexts.

APOE4 models within the occludin and ECM remodelling axis further demonstrated reduced phosphorylation of occludin at Thr residues alongside elevated permeability markers, including fibrinogen, fibronectin, and IgG extravasation in perivascular tissue. Protein kinase C (PKC)η, which regulates occludin phosphorylation and tight junction assembly [57], may therefore represent a key regulatory node in this process.

Nishitsuji et al., [31] demonstrated, using anti-LRP1 treatment in an in vitro BBB model, that PKCη activation was APOE genotype-dependent; inhibition of LRP1 in APOE3 models recapitulated junctional impairment observed in APOE4 conditions, suggesting that LRP1 may function upstream of PKCη-mediated occludin regulation. In APOE4 mice, this pathway appears functionally compromised.

Given that PKC signalling has been shown to increase MMP9 expression through ERK pathway activation and that MMPs disrupt tight junction integrity by degrading occludin and claudin proteins [61], altered PKCη activity in APOE4 contexts may have downstream consequences extending beyond impaired occludin phosphorylation. In addition to reduced phosphorylation-dependent junctional assembly, dysregulated PKC signalling could also potentiate proteolytic degradation of tight junction components via MMP9 upregulation.

PKCη has been linked to AKT and mTOR signalling in models of cellular mechanics [62] and cell proliferation [63], suggesting potential cross-talk between junctional regulation and metabolic signalling networks involving the PI3K/AKT/mTOR pathway. Collectively, these findings support the view that ECM remodelling-related tight junction instability in APOE4 arises from converging MMP9 activity and phosphorylation-dependent signalling perturbations, with potential metabolic contributions rather than a single isolated defect.

Within the third mechanistic axis, insulin/mTOR signalling, several studies reported insulin resistance, impaired glucose uptake (FDG-PET), and reduced cerebral blood volume (CBV) in APOE4 mice – all of which were attenuated by therapeutic administration of rapamycin, an mTOR inhibitor. The mTOR pathway is a recognised regulator of insulin signalling and insulin receptor (INSR) phosphorylation dynamics, and mTOR overactivation is well established as a contributor to insulin resistance [64, 65]. Lin et al. (2014), demonstrated that inhibition of mTOR with rapamycin in APOE4 mice normalised glucose-associated metabolic alterations, supporting a maladaptive role for mTOR hyperactivity in this context.

Genotype-dependent differences in insulin receptor binding and glucose uptake raise an important mechanistic question: whether altered INSR accessibility in APOE4 occurs in parallel with ECM remodelling and junctional destabilisation. Onos et al. (2024), observed reduced glucose uptake in 8-month-old mice, temporally aligning with reports of enzymatic ECM remodelling and compromised basement membrane integrity described above. While these processes were not directly linked experimentally, their co-occurrence supports the possibility of shared upstream dysregulation.

Given that INSR and insulin-like growth factor 1 receptor (IGF1R) share substantial downstream signalling mediators, future studies examining receptor-specific signalling dynamics in APOE-targeted models may clarify whether metabolic and structural BBB perturbations arise from common regulatory mechanisms. Such investigations could help determine whether alterations in cell proliferation or metabolic signalling pathways contribute to the coordinated vascular instability observed [66].

The three mechanistic axes discussed in this systematic review – CypA–MMP9-mediated inflammatory remodelling, mTOR/insulin dysregulation, and occludin-related ECM destabilisation – provide converging evidence for progressive BBB destabilisation in APOE4 contexts. This disruption is characterised by: (1) direct indicators of BBB permeability (e.g., tracer leakage and blood product accumulation); (2) structural features that may drive BBB leakage (e.g., tight junction instability, reduced occludin phosphorylation, focal junctional widening); (3) inflammatory and proteolytic processes that may exacerbate barrier dysfunction (e.g., MMP9 activation, fibronectin deposition); and (4) impaired vascular metabolic regulation (e.g., reduced glucose uptake and CBF deficits).

Future studies should prioritise defining the temporal and causal relationships between these axes, particularly the intersection of metabolic signalling impairment and structural BBB vulnerability. Stage-specific testing using representative markers, such as MMP9 activity (inflammatory modelling), collagen-IV (basement membrane stability), occludin phosphorylation (junctional regulation), PKCη activation (signalling integration), and mTOR pathway activity collectively (metabolic control), may clarify directionality and mechanistic association.

Longitudinal and cell-specific approaches will be essential to determine whether metabolic dysregulation precedes perivascular inflammatory activation, potentially through shared signalling intermediates such as PKCη, or whether these mechanisms of vascular dysregulation occur concurrently.

Future research should also prioritise methodological transparency. Adoption of preclinical reporting standards, such as SYRCLES RoB or ARRIVE guidelines, would improve reproducibility and minimise bias.

Together, these findings support a model of multifactorial BBB vulnerability rather than a single, isolated pathogenic event.

### Limitations

This review has several limitations. First, the number of studies eligible for meta-analysis was relatively small, limiting the statistical power and generalisability of pooled findings. Subgroup analyses were further constrained by methodological heterogeneity, including differences in measurement techniques, biomarkers, and arbitrarily defined age ranges. Additionally, only a small number of studies contributed to the meta-analysis of cerebral blood flow differences (Fig. 2). Although a hierarchical random-effects model using robust variance estimation (RVE) was applied to account for multiple within-study comparisons, the pooled estimates remain derived from a limited study base and should therefore be interpreted with appropriate caution.

Furthermore, many studies received unclear ratings in key domains of the CAMARADES and SYRCLE quality assessments. Details regarding blinding, sample size calculation, and allocation procedures were frequently underreported, limiting assessment of internal validity. These omissions affect not only the reliability of individual studies but also the overall strength of evidence synthesised in this review.

Finally, consistency in genotype comparators would strengthen the interpretability of APOE-targeted studies. In this context, “wild-type” refers to non-humanised C57BL/6 background mice (i.e., mice not carrying APOE-targeted replacement alleles). While wild-type mice provide a useful baseline for transgenic comparisons, *APOE3*-targeted replacement mice represent a more appropriate control when studying humanised *APOE* isoforms, as they isolate isoform-specific effects within the same insertion framework.

It is also important to recognise that targeted replacement of murine *Apoe* with human APOE alleles may itself influence pathological outcomes. Previous studies have demonstrated that APOE insertion can modify disease phenotypes, including delaying or reducing amyloid deposition, compared with models lacking humanised APOE alleles [67, 68]. This underscores the importance of using comparator genotypes within the same targeted replacement framework to distinguish isoform-specific effects from insertion-related influences. Accordingly, direct comparisons between APOE4 and APOE3 knock-in models are essential to isolate pathogenic effects specific to APOE4-related dysfunction and to separate isoform-driven mechanisms from potential insertion-related artefacts.

## Conclusion

In conclusion, this systematic review demonstrates that the APOE4 genotype in mice is associated with compromised blood-brain barrier integrity, reflected by consistent pathogenic alterations in vascular morphology, cerebral blood flow dynamics, and mechanistic pathways linking ECM remodelling, junctional instability and metabolic dysregulation. Preclinical studies using humanised APOE models provide valuable insight into genotype-specific effects and offer a robust framework to inform translational research, including therapeutic development, biomarker discovery, and precision medicine strategies. 

## Supplementary Information

Supplementary Material 1: Table S1. Search terms for MEDLINE, EMBASE, SCOPUS, Web of Science. Table S2. Eligibility criteria.

Supplementary Material 2: Table S3. Inclusion/Exclusion Annotations. Laing

Supplementary Material 3: Table S4. SYRCLE Risk of bias tool assessment by domain

## Data Availability

All data generated or analysed during this study are included in this article and its supplementary information files. The extracted dataset and analysis code that support the findings are available from the corresponding author on reasonable request. The full database search strategies have been deposited on searchRxiv (DOI: 10.1079/searchRxiv.2025.01124). The review protocol was registered with PROSPERO (CRD420251010665): (https://www.crd.york.ac.uk/PROSPERO/view/CRD420251010665).
