# Supplementary material for: Impact of Apolipoprotein E4 on blood-brain barrier integrity in target replacement murine models: a systematic review and meta-analysis
Source: Alzheimers Res Ther. 2026 May 7;18:119. doi: 10.1186/s13195-026-02018-3 (PMC13195987; doi:10.1186/s13195-026-02018-3)
Supplement: Supplementary file 1 — Supplementary Material 1: Table S1. Search terms for MEDLINE, EMBASE, SCOPUS, Web of Science. Table S2. Eligibility criteria. [file 13195_2026_2018_MOESM1_ESM.docx]

**Supplementary Tables**

Supplementary Table 1: Search terms for MEDLINE, EMBASE, SCOPUS, Web of Science

| **Databases** | **Search Terms** |
| --- | --- |
| MEDLINE +  EMBASE | (Apolipoproteins e/ OR Apolipoprotein e3/ OR apolipoprotein e4/) AND (mice/ or mice, transgenic/) AND ((brain/ or blood-brain barrier/) OR (Perfusion/) OR (Cerebrovascular Circulation/)) |
| SCOPUS | (TITLE-ABS-KEY ("apolipoprotein e*" OR "APOE" OR "apolipoproteins e") AND TITLE-ABS-KEY (“mice" OR "transgenic mice" ) AND TITLE-ABS-KEY ( "blood brain barrier" OR "perfusion" OR "cerebrovascular circulation" ) ) AND ( LIMIT-TO ( DOCTYPE , "ar" ) ) |
| Web of Science | ("apolipoprotein e*" OR "APOE" OR "apolipoproteins e" (All Fields)) AND ("mice" OR "transgenic mice" (All Fields)) AND ("blood brain barrier" OR "perfusion" OR "cerebrovascular circulation" (All Fields)) |

Sup. Table 1: Search terms for MEDLINE, EMBASE, SCOPUS, Web of Science

Supplementary Table 2: Eligibility Criteria

|  | **Include** | **Exclude** |
| --- | --- | --- |
| **Population** | - Transgenic murine models with targeted replacement (knock-in) of the endogenous *Apoe* gene with human APOE alleles (e.g., APOE-TR). - EFAD models (5xFAD × APOE-TR). | - Non-humanised Apoe models (e.g., Apoe−/− only). - Other transgenic models without humanised APOE background. - Crosses introducing unrelated genetic modifications (e.g., APP-only knock-in without APOE-TR). - Human studies, non-rodent models, or wild-type-only studies. |
| **Intervention** | - Transgenic murine models in which the murine *Apoe* gene is replaced with human APOE alleles. - In vitro BBB models derived from transgenic APOE murine lines. | - Multi-strain mutant models not centred on humanised APOE. - Studies investigating unrelated pathologies without APOE-targeted replacement background. - Injury models (e.g., traumatic brain injury, cerebral artery occlusion, intracerebral injections). |
| **Comparator** | - Direct comparison between APOE3-targeted replacement and APOE4-targeted replacement mice. | - Studies focused solely on amyloid mechanisms without vascular or BBB comparison. |
| **Outcome** | Quantitative assessments of:   - BBB integrity or vascular morphology (e.g., CD31+, collagen-IV, laminin, pericyte markers). - BBB permeability/leakage (e.g., fibrinogen, IgG, tracer extravasation, Ktrans). - Cerebral blood flow or vascular function. | - Outcomes not related to BBB structure, vascular morphology, permeability, or cerebrovascular function - Molecular or transcriptomic studies without structural or functional BBB measures. - Behavioural-only or cognitive endpoints without vascular assessment. |
| **Study Characteristics** | - Original research articles with quantitative data. | - Review articles, editorials, conference abstracts without primary data - Studies lacking extractable mean ± SEM and sample size data. - Duplicate datasets |

Sup. Table 2: Inclusion and exclusion criteria, specifying criteria used to identify population, interventions and outcome measures
