# Supplementary material for: Impact of Apolipoprotein E4 on blood-brain barrier integrity in target replacement murine models: a systematic review and meta-analysis
Source: Alzheimers Res Ther. 2026 May 7;18:119. doi: 10.1186/s13195-026-02018-3 (PMC13195987; doi:10.1186/s13195-026-02018-3)
Supplement: Supplementary file 3 — Supplementary Material 3: Table S4. SYRCLE Risk of bias tool assessment by domain [file 13195_2026_2018_MOESM3_ESM.docx]

Supplementary Table 4: SYRCLE Risk of Bias Tool assessment by domain.

Risk of bias was evaluated across ten domains: (1) Sequence generation (Was the allocation sequence adequately generated and applied?); (2) Baseline characteristics (Were groups similar at baseline, or adjusted for confounders?); (3) Allocation concealment (Was allocation adequately concealed?); (4) Random housing (Were animals housed randomly during the experiment?); (5) Blinding of caregivers/investigators (Were caregivers/investigators blinded to treatment?); (6) Random outcome assessment (Were animals randomly selected for outcome assessment?); (7) Blinding of outcome assessors (Were outcome assessors blinded?); (8) Incomplete outcome data (Were incomplete data adequately addressed?); (9) Selective outcome reporting (Were reports free from selective outcome reporting?); (10) Other sources of bias (Was the study free of other issues (e.g., contamination, unit‑of‑analysis errors)?). See supplementary material for additional details. **Italicized** studies indicate inclusion in the synthesis. Judgement categories: High Risk, Unclear, Low Risk.

| Study | Selection Bias | | | Performance Bias | | Detection Bias | | Attrition Bias | Reporting Bias | Other Bias |
| --- | --- | --- | --- | --- | --- | --- | --- | --- | --- | --- |
|  | Seq. Gen | Baseline | Alloc. Conceal | Rand. Housing | Caregiver Blinding | Rand. Outcome Assess. | Assessor Blinding | Incomplete Data | Selective Reporting | Other Sources |
| Nishitsuji et al., 2011 | 🟡 | 🟡 | 🟡 | 🟡 | 🟡 | 🟡 | 🟡 | 🟢 | 🟢 | 🟢 |
| *Bell et al., 2012* | 🟡 | 🟢 | 🟡 | 🟡 | 🟡 | 🟡 | 🟡 | 🟢 | 🟢 | 🟢 |
| *Alata et al., 2015* | 🟡 | 🟢 | 🟡 | 🟡 | 🟡 | 🟡 | 🟡 | 🟢 | 🟢 | 🟢 |
| *Thomas et al., 2017* | 🟡 | 🟢 | 🟡 | 🟡 | 🟡 | 🟢 | 🟢 | 🟢 | 🟢 | 🟢 |
| Lin et al., 2017 | 🟡 | 🟢 | 🟡 | 🟡 | 🟡 | 🟢 | 🟡 | 🟢 | 🟢 | 🟢 |
| *Marottoli et al., 2017* | 🟡 | 🟢 | 🟡 | 🟡 | 🟡 | 🟢 | 🟢 | 🟢 | 🟢 | 🟢 |
| *Koizumi et al., 2018* | 🟢 | 🟢 | 🟡 | 🟡 | 🟡 | 🟢 | 🟢 | 🟢 | 🟢 | 🟢 |
| Johnson et al., 2019 | 🟡 | 🟢 | 🟡 | 🟡 | 🟡 | 🟡 | 🟡 | 🟢 | 🟢 | 🟢 |
| *Yamazaki et al., 2020* | 🟡 | 🟢 | 🟡 | 🟡 | 🟡 | 🟡 | 🟡 | 🟢 | 🟢 | 🟢 |
| Ringland et al., 2020 | 🟡 | 🟢 | 🟡 | 🟡 | 🟡 | 🔴 | 🟡 | 🟢 | 🟢 | 🟡 |
| *Lin et al., 2020* | 🟢 | 🟢 | 🟡 | 🟡 | 🟡 | 🟡 | 🟡 | 🟢 | 🟢 | 🟢 |
| *Montagne et al., 2021* | 🟢 | 🟢 | 🟢 | 🟡 | 🟢 | 🟢 | 🟢 | 🟢 | 🟢 | 🟢 |
| Yamazaki et al., 2021 | 🟢 | 🟢 | 🟡 | 🟡 | 🟡 | 🟢 | 🟡 | 🟢 | 🟢 | 🟢 |
| Rhea et al., 2021 | 🟢 | 🟢 | 🟡 | 🟡 | 🟡 | 🟢 | 🟡 | 🟢 | 🟢 | 🟢 |
| Barisano et al., 2022 | 🟢 | 🟢 | 🟢 | 🟡 | 🟢 | 🟢 | 🟢 | 🟢 | 🟢 | 🟢 |
| Jackson et al., 2022 | 🟡 | 🟢 | 🟡 | 🟡 | 🟡 | 🟡 | 🟡 | 🟢 | 🟢 | 🟢 |
| Yanckello et al., 2022 | 🟢 | 🟢 | 🟡 | 🟢 | 🟡 | 🟢 | 🟡 | 🟢 | 🟢 | 🟢 |
| Bonnar et al., 2023 | 🟡 | 🟢 | 🟡 | 🟡 | 🔴 | 🟡 | 🟢 | 🟢 | 🟢 | 🟢 |
| Onos et al., 2024 | 🟢 | 🟢 | 🟢 | 🟢 | 🟢 | 🟢 | 🟢 | 🟢 | 🟢 | 🟢 |
| Anderle et al., 2025 | 🟢 | 🟢 | 🟡 | 🟡 | 🟡 | 🟢 | 🟢 | 🟢 | 🟢 | 🟢 |
| Bhattarai et al., 2025 | 🟡 | 🟢 | 🟡 | 🟡 | 🟡 | 🟡 | 🟢 | 🟢 | 🟢 | 🟡 |
